# Sulfide Intrusion and Detoxification in the Seagrass *Zostera marina*

**DOI:** 10.1371/journal.pone.0129136

**Published:** 2015-06-01

**Authors:** Harald Hasler-Sheetal, Marianne Holmer

**Affiliations:** Department of Biology, University of Southern Denmark, Odense, Denmark; Auckland University of Technology, NEW ZEALAND

## Abstract

Gaseous sulfide intrusion into seagrasses growing in sulfidic sediments causes little or no harm to the plant, indicating the presence of an unknown sulfide tolerance or detoxification mechanism. We assessed such mechanism in the seagrass *Zostera* marina in the laboratory and in the field with scanning electron microscopy coupled to energy dispersive X-ray spectroscopy, chromatographic and spectrophotometric methods, and stable isotope tracing coupled with a mass balance of sulfur compounds. We found that *Z*. *marina* detoxified gaseous sediment-derived sulfide through incorporation and that most of the detoxification occurred in underground tissues, where sulfide intrusion was greatest. Elemental sulfur was a major detoxification compound, precipitating on the inner wall of the aerenchyma of underground tissues. Sulfide was metabolized into thiols and entered the plant sulfur metabolism as well as being stored as sulfate throughout the plant. We conclude that avoidance of sulfide exposure by reoxidation of sulfide in the rhizosphere or aerenchyma and tolerance of sulfide intrusion by incorporation of sulfur in the plant are likely major survival strategies of seagrasses in sulfidic sediments.

## Introduction

Seagrasses colonize coastal sediments characterized by low oxygen concentrations and high concentrations of toxic, reduced substances such as iron, manganese, and sulfide [1]. The effect of sulfide on growth and health of seagrasses is particularly puzzling. Sulfide is toxic to eukaryotic cells including seagrass cells even at concentrations as low as 1 to 10 μmol L^-1^ [2,3,4]. However, seagrasses can thrive in sediments with sulfide concentrations in the millimolar range [5]. The Mediterranean seagrass *Posidonia oceanica* (Linnaeus) Delile tolerates only low concentrations of pore water sulfide [6], whereas *Zostera marina* Linnaeus, the most common seagrass in temperate waters [7], can tolerate high concentrations of up to 4 mmol L^-1^ sulfide both *in situ* and *in vitro* [6,8,9].

Field and laboratory studies show that oxygen and sulfide dynamics in the water column, sediment, and plant tissues are key factors for seagrass growth, colonization, and survival [8,10,11]. Seagrasses avoid root anoxia and sulfide intrusion by leakage of oxygen from the roots (radial oxygen loss, ROL) [12,13]: photosynthetically derived oxygen diffuses via the aerenchyma to the respiring underground tissues [14], where it leaks out through the root tips to maintain an oxic rhizosphere [15]. Remarkably, the zone behind the root-tip region contains barriers to avoid ROL and sulfide intrusion [15,16]. Molecular oxygen derived from ROL probably also oxidizes toxic compounds such as sulfide to non-toxic compounds such as sulfate or elemental sulfur (S^0^), before reaching the root surfaces [15,17,18]. Oxidation of sulfide occurs both inside the plant in the aerenchyma [13] and externally in the rhizosphere [15,19,20]. In the dark, the photosynthetic oxygen pool in the aerenchyma is depleted in a few minutes [21] and the plant internal oxygen partial pressure is maintained by oxygen diffusion from the water column [10]. Thus, darkness combined with events of hypoxia or anoxia in the water column can lead to sulfide intrusion in the plants [13]. When oxygen returns into the plant, intruded sulfide can be oxidized: this has been observed in *Z*. *marina*, where gaseous sulfide in the aerenchyma disappears as oxygen is produced when photosynthesis commences in the morning [10,13].

In addition to microelectrode and optode studies, intrusion of sediment sulfide into seagrass tissues has been demonstrated by studies using stable sulfur isotopes [5]. High sulfur accumulation caused by sulfide intrusion has been found in most seagrasses studied to date and is correlated with environmental factors such as sediment sulfide concentration, water-column hypoxia, and shading [11,22,23]. In *Z*. *marina*, sulfur accumulates as S^0^ and other yet-unidentified sulfur compounds [11,24]. These unknown sulfur compounds accumulate to a high extent in rhizomes exposed to sulfide intrusion and, together with S^0^, are speculated to play a role in sulfide detoxification in the plant [6,11]. So far, S^0^ has been found and located by scanning electron microscopy with energy dispersive X-ray spectroscopy (SEM-EDX) in the vascular tissue of terrestrial plants [25]; in seagrasses, S^0^ has been found in underground tissues [22] but not yet localized. Despite these efforts, the mechanisms and metabolic pathways that cause S^0^ formation and accumulation remain unclear [26], as do their consequences for the seagrass.

Sediment sulfide intrudes in gaseous form via the roots of the plant [13] due to the membrane permeability of sulfide [27,28], and spreads by gas diffusion through the aerenchyma to the rhizome [21]. Once intruded, sulfide is available for enzymatic assimilation, possibly into thiol compounds (e.g., cysteine and glutathione) as occurs in wetland [29] and terrestrial [30,31] plants. Both terrestrial and wetland plants can incorporate and detoxify considerable amounts of sulfide, taken up by either the leaves or the underground tissues, resulting in elevated levels of sulfur compounds such as sulfate, thiols, and total sulfur in plant tissue without alteration of growth rates [31]. When sulfide uptake in terrestrial plants is foliar, the sulfur compounds are deposited in only the tissues exposed to sulfide and not translocated to other, more distal plant tissues [32]. However, soil or sediment sulfide uptake via roots typically results in higher accumulation of sulfur in the rhizome than the roots [11,31].

Terrestrial plants (e.g., *Alium cepa* (Linnaeus) and *Brassica oleracea* (Linnaeus)) can utilize gaseous sulfide as a source of sulfur, decreasing sulfur demand [32–34]. However, sulfate, which is considered to be the main source of sulfur nutrition, is mainly taken up by the roots, transported and stored in vacuoles throughout the plant [35,36]. In cases of sulfate deprivation in terrestrial plants, foliar uptake of sulfide can be an important sulfur source [32–34]. In contrast to terrestrial systems, the coastal marine environment offers three major sulfur sources for seagrasses: sulfate in the pore water, sulfate in the water column—both have roughly the same sulfate concentration and isotopic composition (γ^34^S around +20‰)—and sediment sulfide, which has a distinctly different isotopic composition (γ^34^S around −20‰) [37,38]. However, the contribution of each sulfur pool to sulfur nutrition in seagrasses has not been explored.

This study aims to characterize yet-unknown sulfur compounds and their origins in *Z*. *marina* subject to sulfide intrusion. We hypothesized that intruding sediment sulfide is metabolized and/or precipitated, and that sulfide is detoxified to non-toxic compounds inside *Z*. *marina*.

## Material and Methods

### Field study

We conducted a field study to assess the natural levels of sulfide intrusion in *Z*. *marina* and therefore collected *Z*. *marina* plants at a water depth of one meter from three intact seagrass meadows at Svenstrup Strand (+55°28'07", +9°45'18"), Kertinge Nor (+55°26'55", +10°33'27"), and Dalby Bay (55°31'07", +10°37'05"), Denmark, during July and August 2012. Plants were collected haphazardly by harvesting turfs (n = 5) with intact ramets. The salinity was 18. The water temperature was 18.6 ± 0.3°C in July and 18.1 ± 0.4°C in August, respectively.

The plants were gently and thoroughly rinsed with sea water and deionized water to remove sediment, root precipitates, and salts. Macroscopic epiphytes were removed by hand and plants were separated into the two youngest leaves, rhizome segments, and root bundles, then snap-frozen in liquid nitrogen. All samples were frozen (-80°C) until lyophilization for 48 h and homogenized in a ball mill before further processing. Svenstrup strand, Kertinge Nor and Dalby bay are public area, and in Denmark no specific permission is required for scientific research in this public areas. No endangered or protected species were involved in the study. This article does not report studies with human or animal subjects.

### Mesocosm study

To determine the effect and fate of sulfide in *Z*. *marina*, we performed a mesocosm experiment in which we caused sulfide intrusion by artificially increasing pore water sulfide concentrations and measured its effects on plant sulfur compounds.

We collected medium sand sediment with low organic matter content and low iron pools [39] adjacent to a seagrass meadow at Svenstrup Strand in October 2012 at a water temperature of 15.2°C. The sediment was passed through a 1 mm sieve and placed in pots (11 cm in diameter and 12 cm height). To enhance sulfate reduction and hence sulfide concentration in the rhizosphere, we enriched half of the pots with caramelized glucose (720 g m^-2^; high sulfide, HS) at a sediment depth of 5 cm. The other pots acted as controls (C). After 10 days, apical shoots were collected from Svenstrup Strand as described previously. Senescent leaves, rhizome parts older than five internodes, and epiphytes were gently removed. Ten shoots were transplanted into each pot, yielding a shoot density of 1052 shoots m^-2^ (reflecting field densities) and placed in a mesocosm filled with seawater. Two shoots of each replicate were marked to estimate leaf growth, as described by Sand-Jensen [40]. The salinity (18) and temperature (15°C) were kept constant and half of the water was exchanged every week. Artificial illumination (Phillips SONT-T Agro 400W) was set to a diel cycle of 12:12 h at an intensity of 180–200 μmol photons m^-2^ s^-1^ at canopy level. The water column was aerated during the light period, but hypoxic during darkness to achieve sulfide intrusion following the method of Mascaro et al. [11], who ceased aeration during darkness resulting in oxygen levels at canopy height of around 40% of air saturation at the end of the dark cycle. After three weeks of exposure, we harvested the plants, separated them into leaves, rhizome, and root tissue, and preserved them as described above.

#### Plant sulfur extraction and analysis

Sulfate in plant tissue (leaf, rhizome, and root) was measured in the supernatant after a hot water extraction (20 mg plant tissue in 2 mL H_2_O, 95°C for 2 h, then centrifuged 5 min at 16000 × g, filtered through 0.45 μm pore size) on an ion chromatograph (DIONEX ICS-1500, DIONEX, Sunnyvale, CA, USA; IonPac AS22, 4.5 mM Na_2_CO_3_/1.4 mM NaHCO_3_). The remaining pellet was snap-frozen in liquid nitrogen and later lyophilized, and represents the water insoluble fraction (IF). S^0^ in plant tissues (10 mg extracted in 5 mL methanol for 24 h in darkness) was measured according to the method of Zopfi et al. [41] on an UV-RPHPLC (Agilent 1100 Series HPLC with UV detector (265 nm), Agilent Technologies Inc., Santa Clara, CA, USA). Concentrations of thiols (soluble, non-protein organo-sulfur compounds characterized by a chemical formula of R-SH) were estimated spectrophotometrically using a modified protocol of de Kok et al. [42] and lyophilized instead of fresh tissue. We tested the modification for glutathione recovery and found no significant differences between lyophilized and fresh tissues (data not shown). Samples of the whole plant organs (bulk S), the IF of the different tissues, and plant internal sulfate were analyzed for total sulfur (TS) and stable sulfur isotope ratio (δ^34^S) by elemental analyzer combustion continuous flow isotope ratio mass spectroscopy (EA-C-CF-IRMS; Thermo Scientific Delta V Advantage plus Flash EA 1112, Thermo Fisher Scientific Inc., Waltham, MA, USA). Plant internal δ ^34^S-sulfate and TS were measured as δ ^34^S and TS of BaSO_4_ after a hot water extraction (50 mg dry weight (DW) mL^-1^) for 1 h at 95\°C followed by precipitation with 1 M BaCl_2_ added to the filtered (0.45 μm) supernatant. The δ^34^S signal of the organic sulfur (TS-(sulfate+S^0^)) fraction was calculated according to the following isotope mass balance (Eq 1):

$$\delta^{34}{\text{S}}_{o}=\frac{\delta^{34}{\text{S}}_{b}\times {\text{mS}}_{b}-{\text{mS}}_{i}\times \delta^{34}{\text{S}}_{i}-{\text{mS}}_{s}\times \delta^{34}{\text{S}}_{s}}{{\text{mS}}_{o}-{mS}_{s}}$$ (1)

where δ^34^S represents the sulfur isotope ratio, m the mass; *o* the organic sulfur, *b* the bulk sulfur value, *s* the sulfate value, and *i* the IF. The mass of organic sulfur was assessed by the difference between bulk sulfur and IF + sulfate.

#### Localization of sulfur

The distribution of sulfur inside the aerenchyma and in parenchymatous tissue was obtained by SEM-EDX as described by Williams et al. [43]. Cross (1 mm thick) and longitudinal sections (1–4 mm long) through the two youngest root bundles of the plants from the mesocosms were obtained with a dichloromethane-washed scalpel. The sections were mounted on an adhesive carbon disc on an aluminum stub. Instant cryofixation was applied to avoid wash out of S^0^ [44–46] followed by lyophilization for 12 h [43]. SEM-EDX analysis was done without coating to avoid alteration of the elemental composition by the coating material. SEM-EDX spectra were measured on a PHILIPS XL 20 using ZAF quantification under 20 kV to assess the relative abundance of sulfur in the sample using the Gensis Software (EDAX, San Francisco, USA).

#### Sediment and water analysis

Pore water, extracted by rhizons (5 cm, rhizosphere.com, Wageningen, NL) utilizing vacuetts, and the water column were sampled weekly. Five milliliter pore water was immediately frozen for later analysis of nutrients and one milliliter immediately preserved with 100 μL 1 M zinc acetate for measuring sulfide concentrations [47]. Pore water sulfate was measured on an ion chromatograph (DIONEX, IonPac AS22). Ammonium [48] and phosphate [49] were analyzed spectrophotometrically. The δ^34^S of pore water and water column sulfate was measured as δ^34^S of BaSO_4_, as described above. To assess the isotopic signal of sediment, sediment cores (2.6 cm in diameter and 5 cm in depth) were taken at the start and the end of the mesocosm experiment. The sediment was preserved in 1 M zinc acetate (vol:vol) and frozen until later analysis. The sediment was distilled following the two-step method of Fossing and Jørgensen [50], where the first step extracts acid-volatile sulfide (AVS), consisting of FeS and H_2_S, and the second step chromium-reducible sulfur (CRS), consisting of Fe_2_S and S^0^. Sulfide was precipitated with Ag_2_S and measured for δ^34^S by EA-C-CF-IRMS.

#### Stable sulfur isotope tracing of sulfur compounds

We expected δ^34^S of sediment sulfide to increase following enrichment with glucose, caused by decreased discrimination of ^34^S at high rates of sulfate reduction; in contrast, δ^34^S of pore water and water column sulfate were not expected to be affected [6,11,51]. The difference in δ^34^S between HS and C is given as Δδ^34^S. Further, we used Δδ^34^S-sulfide between HS and C to trace which plant sulfur compounds originated from sediment sulfide. Another advantage of applying the Δδ^34^S is that all temporal variations in δ^34^S-sulfur sources during the experiment—e.g., diurnal variations that are the same for HS and C—are removed. This enabled us to exclusively trace the fate of intruding sediment sulfide in the sulfur compounds of seagrass tissues.

### Statistical procedures

All figures were plotted in GraphPad Prism version 6 for Mac (GraphPad Software, La Jolla, CA, USA). Statistical analyses were conducted in JMP, Version 10 (SAS Institute Inc., Cary, NC, USA, 1989–2013). Analysis of variance (ANOVA) and the t-test were used if test assumptions were fulfilled. For results of ANOVA that showed significant effects, the Tukey’s honestly significant difference *post-hoc* procedure was used to determine significantly different means. If ANOVA assumptions were not achieved then non-parametric tests (Kruskal-Wallis and *post-hoc* Dunn’s test) were applied. Analysis of covariance (ANCOVA) was used to compare slopes of two or more regression lines if test assumptions were fulfilled. Data were adjusted for outliers using the Grubbs’ test. All mean values are presented with standard error of mean (mean ± SEM).

## Results

### Sediment sulfur analysis

Glucose enrichment of the sediments (HS) triggered a higher pore water sulfide concentration: 2.8 ± 0.4 mmol L^-1^ compared with 0.9 ± 0.3 mmol L^-1^ in C. In contrast, pore water sulfate, ammonium, and phosphate concentrations did not respond to glucose enrichment (S1 Table), indicating no influence of nutrients on seagrass performance in this study. As expected, the δ^34^S of sediment sulfide shifted in the glucose treatment (p < 0.0001; δ^34^S AVS: 6.9 ± 1.5‰ for HS and −16 ± 1.3% for C; δ^34^S CRS: −12.7 ± 1.5‰ for HS and −22.6 ± 0.5‰ for C), reflecting the expected increase in δ^34^S due to glucose enrichment. Of the three possible sulfur sources for seagrasses, the δ^34^S values of two—the pore water sulfate (20.9 ± 0.8‰) and the water column sulfate (20.4 ± 1.2‰)—did not respond to treatment (p > 0.05), whereas the third, the δ^34^S of sediment sulfide (AVS), shifted (p < 0.0001) by a Δδ^34^S of 22.9 ± 0.4‰. Consequently, every observed change between HS and C in the δ^34^S (Δδ^34^S) of plant sulfur compounds must have originated from intrusion of sediment sulfide.

### Plant sulfur analysis

Plants in both treatment groups showed similar growth rates (HS: 16 ± 1 mm m^-1^ d^-1^ vs. C: 19 ± 2 mm m^-1^ d^-1^) (p > 0.05) and survival rates (83 ± 3.9% for HS and 95 ± 2.7% for C; p > 0.05), although there was a trend for lower values in the HS treatment group.

**Fig 1:**
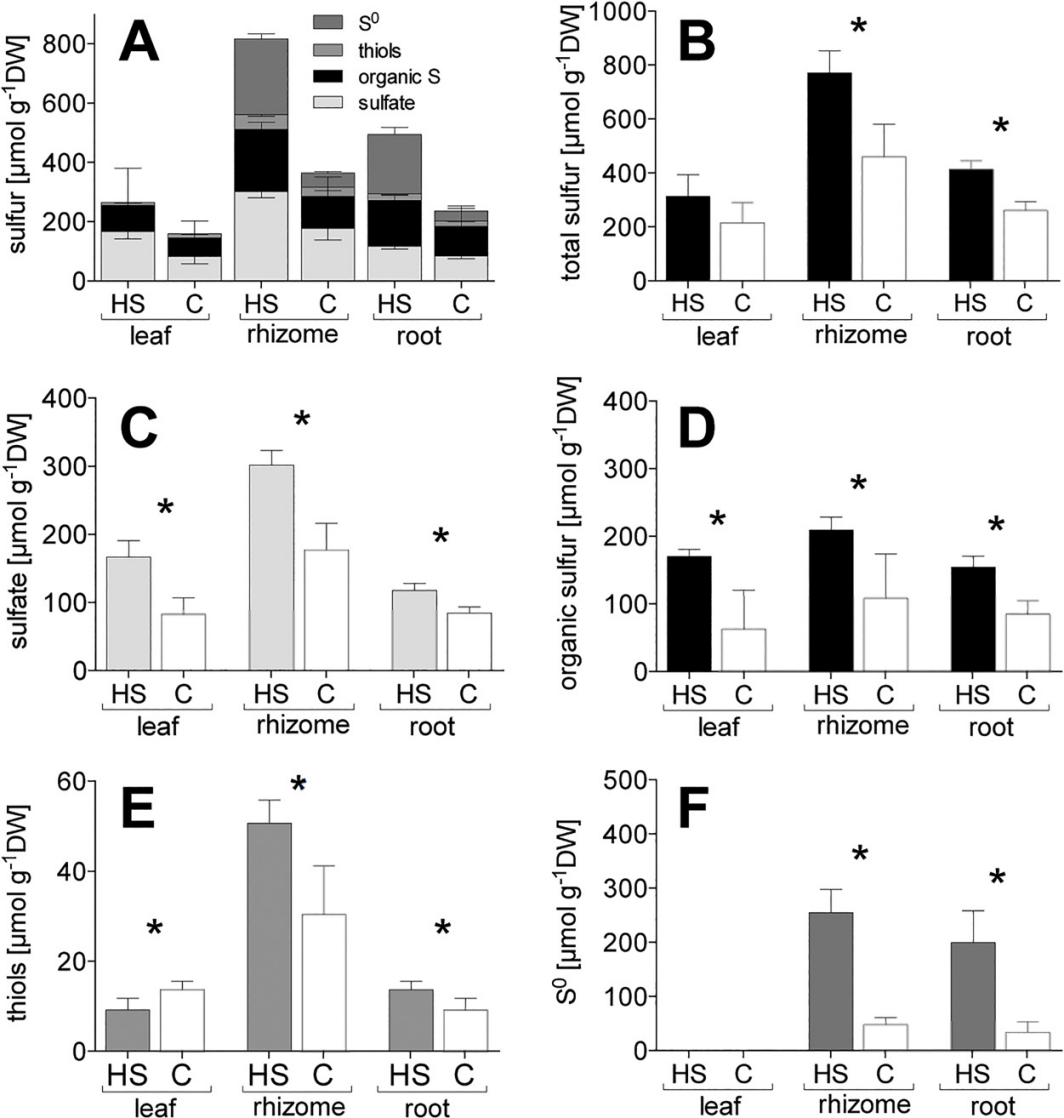
Sulfur budget of different fractions of *Zostera marina in vitro* (A) stacked values of all fractions, (B) total sulfur, (C) sulfate, (D) organic sulfur, (E) thiols, (F) S^0^. Values represent mean ± SEM; n = 6. C, control; DW, dry weight; HS, high sulfide. Columns connected by the asterisk indicate significant intra-tissue differences (ANOVA and Tukey’s test, p < 0.05).

High sediment sulfide levels (HS) caused an accumulation of sulfur compounds in the roots, rhizomes, and leaves of *Z*. *marina* (Fig 1A): (i) The total sulfur content was highest in the rhizome followed by the roots, whereas there was no increased accumulation in the leaves (Fig 1B). (ii) The levels of sulfate and organic sulfur increased in all three tissues (Fig 1C and 1D). (iii) The levels of thiols increased in underground tissues and decreased in the leaves (Fig 1E). (iiii) There was high accumulation of S^0^ in the underground tissues and, remarkably, a total absence in leaves (Fig 1F). There are methodological problems for the detection of S^0^ in the leaves by UV-RPHPLC due to interference of chlorophyll with the S^0^ signal [22]. To overcome this issue, we assessed the amount of sulfur in the IF, which includes S^0^, and found no accumulation of sulfur in the leaves of C or HS (data not shown). However, the sulfur in the IF of underground tissues is exclusively S^0^. Therefore, we refer to δ^34^S-S^0^ when presenting the δ^34^S of the IF.

**Fig 2:**
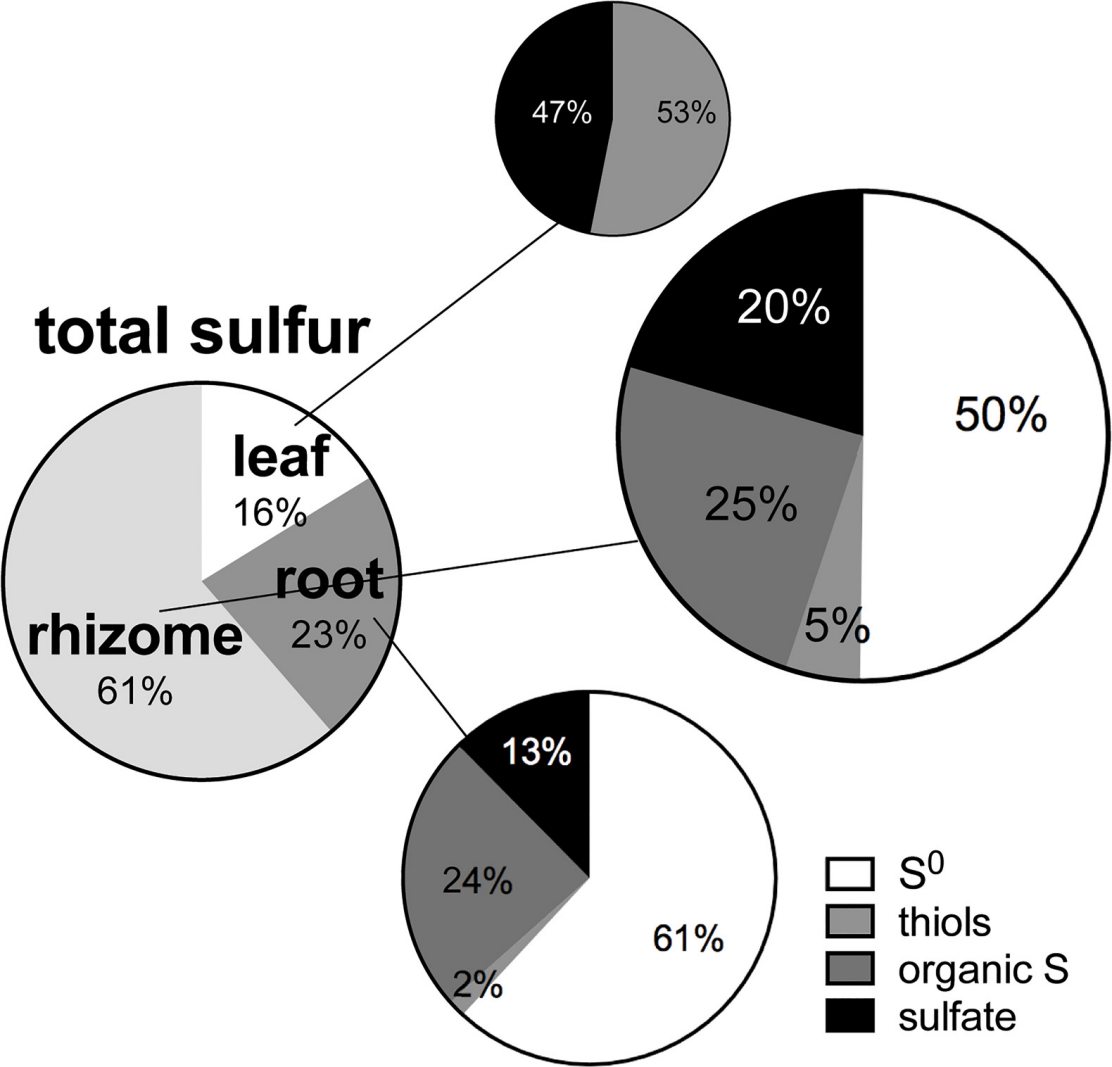
Relative net accumulation of total sulfur in *Zostera marina* (HS minus C) including the assemblage total sulfur composition of sulfur compounds in the different tissues (n = 6).

The net accumulation of total sulfur and sulfur compounds in HS plants (HS minus C) shows a major sulfur accumulation in underground tissues (84% in roots + rhizomes, 16% in leaves) (Fig 2) with a prominent share of S^0^ (45%). The proportion of S^0^ of TS in the rhizome was 61% and in the roots 50%.

**Fig 3:**
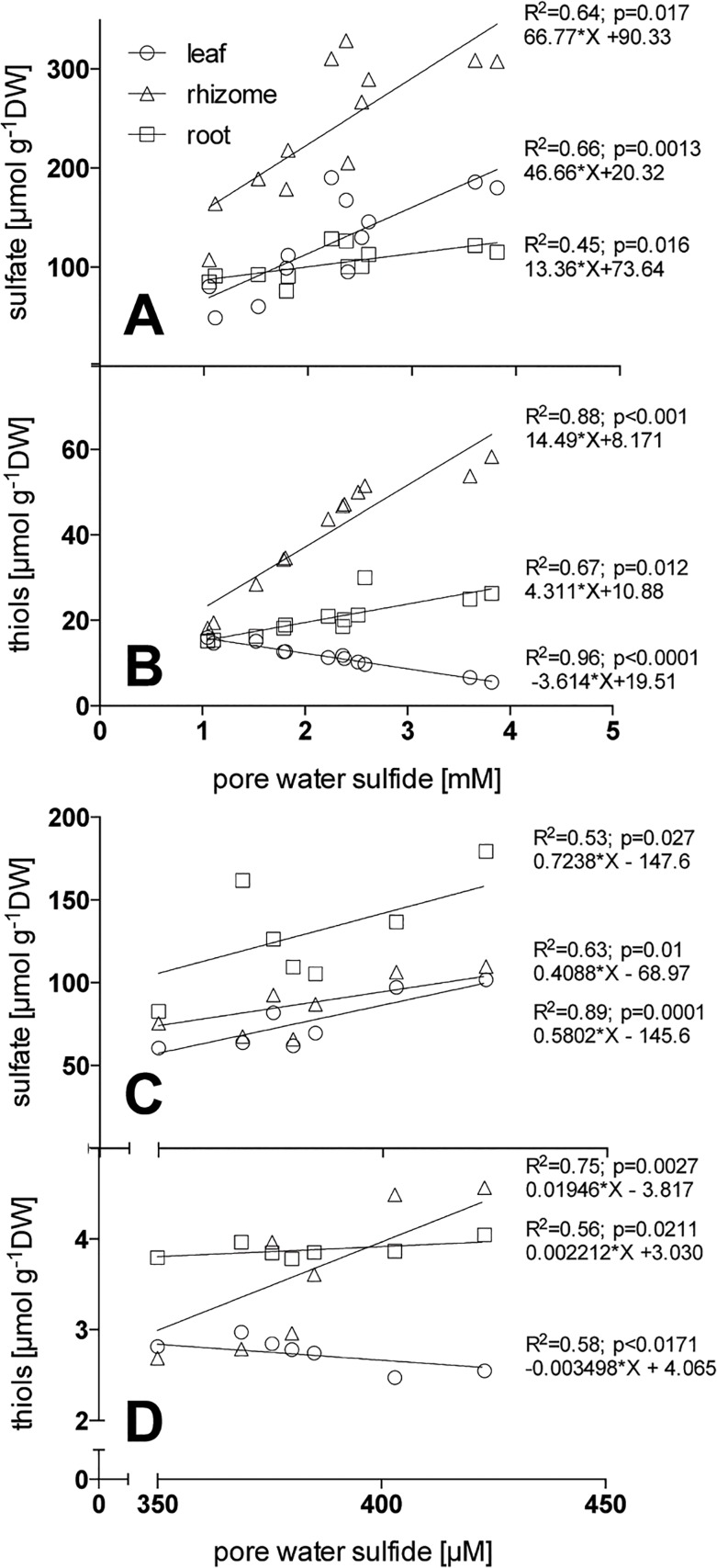
Linear regressions of pore water sulfide and *Zostera marina* tissue sulfate (A, C) and thiol concentration (B, D) from plants exposed to high pore water sulfide concentrations in experimental conditions (A, B) and from field plants (C, D); n = 12 for A, B and 9 for C, D. DW, dry weight.

Plant sulfate levels were driven by pore water sulfide concentrations, showing a positive linear relationship: slopes were similar for roots and rhizomes but steeper for leaves (Fig 3A). Thiols in the plants were primarily influenced by pore water sulfide concentrations; related positively in underground tissues (with a steeper slope for the rhizome) and negatively in leaves (Fig 3B).

**Fig 4:**
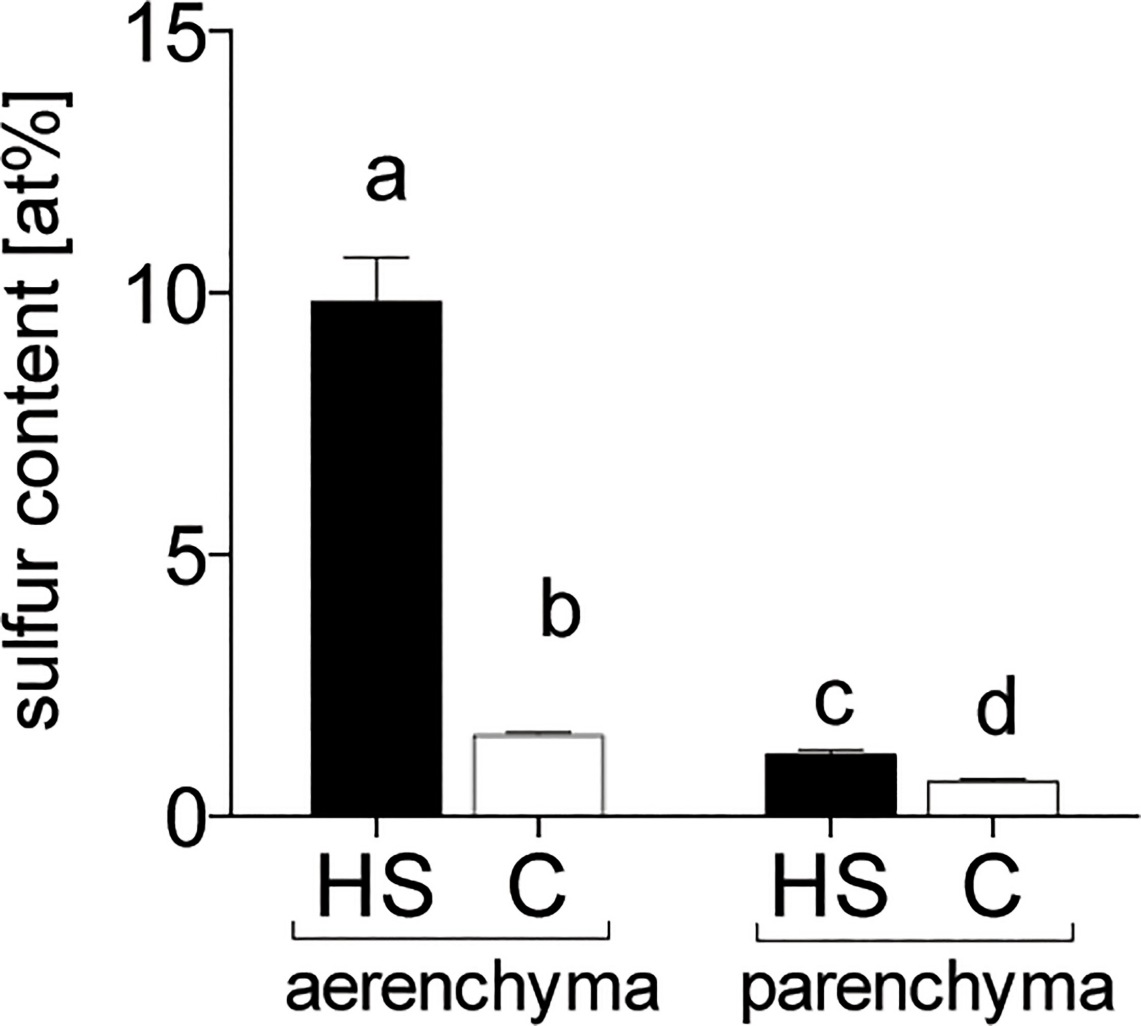
Sulfur content in atomic-percent [at%] by SEM-EDX in *Zostera marina* roots exposed to sulfide intrusion. Letters indicate significant differences (Dunn's test p < 0.05); n = 48. HS = high sulfide; C = control.

SEM-EDX analysis revealed sulfur accumulation to be higher in the root arenchyma than the parenchyma (Fig 4). HS treatment led to 6.4-fold higher sulfur content in aerenchymatous tissue and 1.7-fold higher in parenchymatous tissue compared with C.

### Stable isotope results

High pore water sulfide concentrations consistently increased the δ^34^S values of all sulfur compounds in the plants (Table 1), with higher values in the underground tissues than the leaves.

**Table 1:** Sulfur isotopic composition (δ^34^S, mean ± SEM) in per mille [‰] of bulk sulfur, insoluble sulfur compounds (S^0^), organic sulfur, and sulfate in different *Zostera marina* tissues cultivated under high sediment sulfide concentrations (HS) and control conditions (C); n = 6.

Treatment	Tissue	δ^34^S [‰]			
		bulk S	S^0^	organic sulfur	sulfate
**HS**	*Leaf*	3.61 ± 0.62^*A,b^	0.24 ± 0.49^*B,c^	3.2 ± 0.67^*A,b^	4.47 ± 0.28^*A,a^
	*Rhizome*	8.37 ± 0.99^*A,a^	6.07 ± 0.61^*A,a^	7.88 ± 1.06^*A,a^	5.46 ± 0.3^*A,a^
	*Root*	7.24 ± 0.99^*A,a^	2.77 ± 0.67^**B,b^	6.75 ± 1^*A,a^	5.22 ± 0.27^*AB,a^
**C**	*Leaf*	-2.12 ± 0.85^*A,a^	-4.03 ± 0.61^*A,a^	-1.9 ± 0.88^*A,a^	-2.76 ± 1.25^*A,a^
	*Rhizome*	-7.29 ± 0.5^*AB,b^	-8.65 ± 0.51^*B,b^	-7.07 ± 0.51^*AB,b^	-5.11 ± 0.93^*A,a^
	*Root*	-5.6 ± 0.68^*AB,b^	-7.98 ± 0.61^*B,b^	-5.35 ± 0.7^*A,b^	-3.78 ± 0.49^*A,a^

Note that S^0^ was assessed from insoluble sulfur compounds. Asterisks indicate significant differences between HS and C. Capital letters indicate significant differences between compounds within a tissue; lower case letters indicate significant differences between the tissue types in a specific compound. ANOVA and post hoc Tukey’s test; p < 0.05.

**Fig 5:**
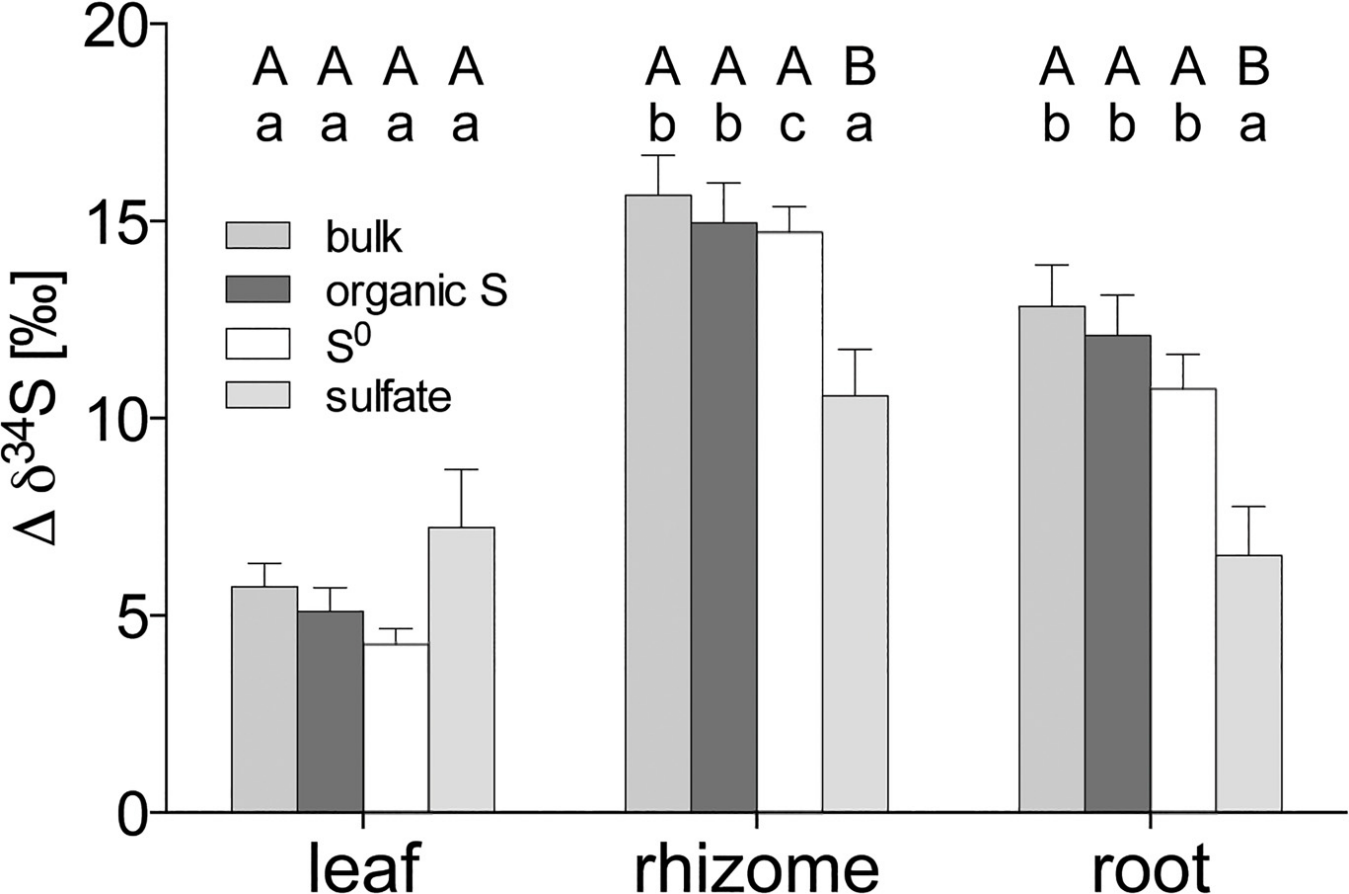
Sulfur isotopic response in sulfur compounds in *Zostera marina* upon sulfide intrusion. Shift of average δ^34^S values (± SEM) in per mille [‰] of bulk sulfur and different sulfur compounds from *Zostera marina* between high sulfide concentrations (HS) and control conditions (C); n = 6. Capital letters indicate significant differences between compounds in a tissue and lower case letters indicate differences between tissues for a specific compound (ANOVA and Tukey’s test, p < 0.05).

The Δδ^34^S of all plant sulfur compounds except sulfate were higher in the underground tissues than in the leaves (Fig 5). Δδ^34^S-sulfate did not vary between tissues. In the leaves, all sulfur compounds shifted to the same extent, whereas in the underground tissues, Δδ^34^S of bulk sulfur, organic sulfur, and S^0^ increased more than sulfate (Fig 5), indicating that sulfide is a precursor to these compounds. Δδ^34^S-sulfide explains to a high extent the increased Δδ^34^S levels of all sulfur compounds in underground tissues, and organic sulfur and sulfate in the leaves, respectively (S1 Fig, p < 0.01, R^2^ > 0.89); with similar slopes for the sulfur compounds within a tissue (p > 0.05; pooled slopes: leaf = 1.72, rhizome = 1.67, root = 1.62). In contrast, the Δδ^34^S of the other sulfur sources—pore water sulfate and water column sulfate—did not explain the increase in Δδ^34^S at all (linear regression; p > 0.05).

### Field study

The pore water sulfide concentrations ranged from 321 to 423 μmol L^-1^ at all sites, which are moderately high levels for sediments in coastal Denmark during the summer months. The δ^34^S value of sediment sulfide was not significantly different between sites and varied between −24.9‰ and −12.8‰ for AVS and −30.3‰ and −22.8‰ for CRS.

**Fig 6:**
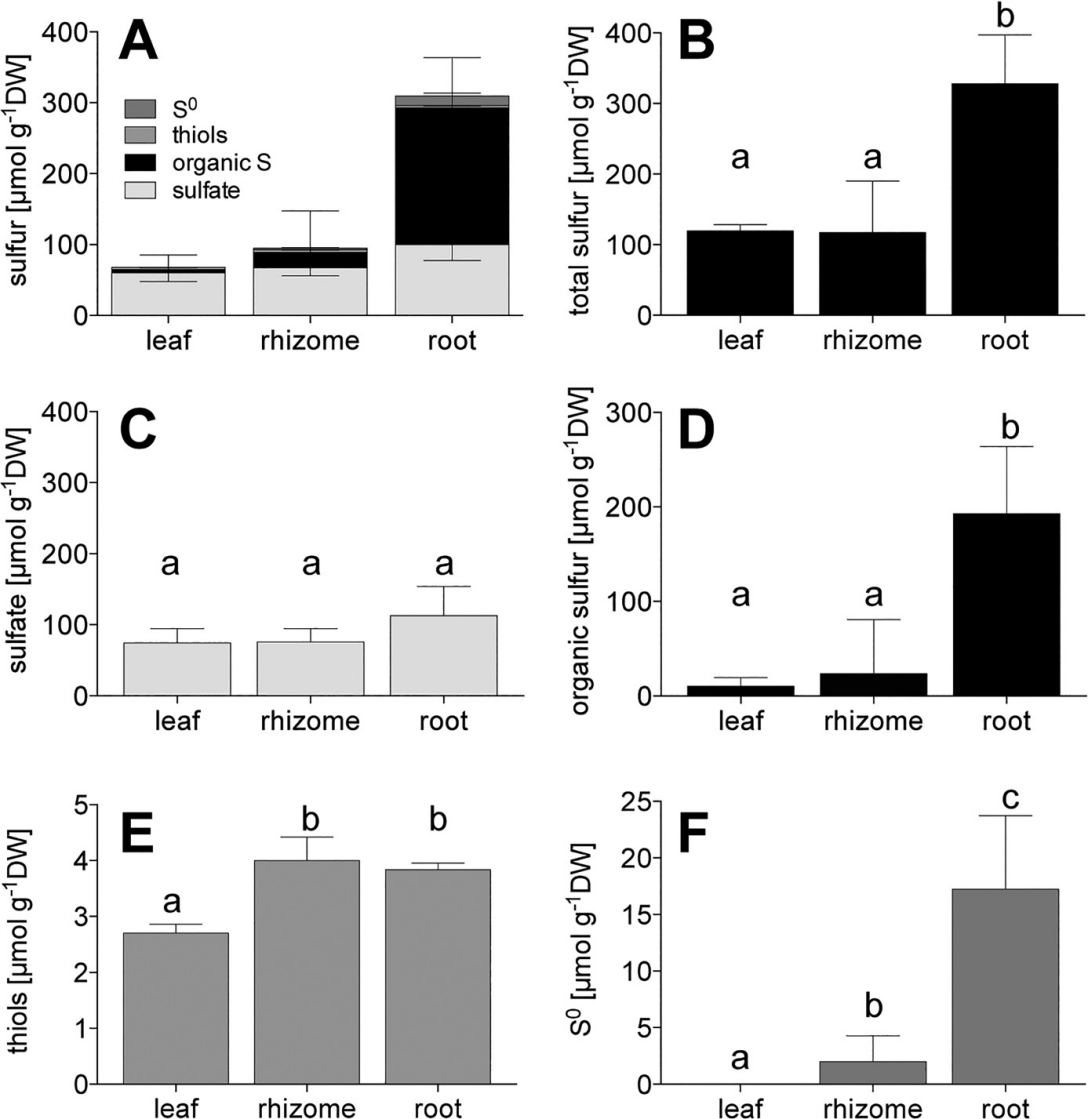
Sulfur budget of different fractions of *Zostera marina in situ* (A) stacked values of all fractions, (B) total sulfur, (C) sulfate, (D) organic sulfur, (E) thiols, (F) S^0^. Values represent mean ± SEM. Lower case letters indicate differences between tissues (ANOVA and Tukey’s test, p < 0.05); n = 12.

The highest accumulation of sulfur in field plants was found in the roots (Fig 6A and 6B), primarily caused by significantly higher accumulation of sulfate and organic sulfur (Fig 6D and 6F) (p < 0.0001). The levels of other sulfur compounds (thiols and S^0^) did not vary between tissues and sites (Fig 6E and 6F). The sulfate concentration in the plant tissues was positively linearly related to the pore water sulfide concentration, with similar slopes for root, rhizome, and leaves (p > 0.05; pooled slope = 0.57) (Fig 3C) and independent of site. Thiols in the plant were linearly related to pore water sulfide concentrations, positively in the underground tissues (with a steeper slope for the rhizome) and negatively in the leaves (Fig 3B). The δ^34^S of bulk sulfur, organic sulfur, and S^0^ in plants were most negative in the roots followed by rhizomes and leaves with the exception of δ^34^S-sulfate, which did not vary between tissues (Table 2) (p < 0.05).

**Table 2:** Sulfur isotopic composition (δ^34^S, mean ± SEM) in per mille [‰] of bulk S, insoluble sulfur compounds (S^0^), organic sulfur, and sulfate in different *Zostera marina* tissues *in situ*; n = 6.

Tissue	δ^34^S [‰]			
	bulk S	S^0^	organic sulfur	sulfate
*Leaf*	1.71 ± 0.86^A,a^	1 ± 0.98^A,a^	3.02 ± 1.25^A,a^	2.15 ± 0.55^A,a^
*Rhizome*	0.61 ± 1^AB,a^	-2.17 ± 0.82^B,a^	5.01 ± 1.38^C,a^	2.14 ± 0.27^A,a^
*Root*	-10.24 ± 0.76^A,b^	-9.11 ± 0.96^A,b^	-11.27 ± 1.4^A,b^	0.89 ± 0.73^B,a^

Note that S^0^ was assessed from insoluble sulfur compounds. Capital letters indicate significant differences between compounds within a tissue; lower case letters indicate significant differences between the tissue types in a specific compound. ANOVA and post hoc Tukey’s test; p < 0.05.

## Discussion

### Identification of sulfur compounds

High levels of sulfide intrusion lead to increased sulfur pools in *Z*. *marina*, consisting of several sulfur compounds including S^0^, sulfate, organic sulfur, and thiols. S^0^ accumulated inside the aerenchyma, a pattern that was probably caused by chemical reoxidation of toxic sulfide and its precipitation as non-toxic S^0^ on the inner wall of the aerenchyma. This mechanism is indicated by high and similar accumulation of sulfur on the inner wall of the root aerenchyma (detected by SEM-EDX) and S^0^ in underground tissues exposed to sulfide intrusion. Both increased at similar rates—6.4-fold in the aerenchyma and 6.6-fold in the roots, respectively, but only 1.7-fold in the parenchyma. This is similar to the 1.4-fold increased sulfate accumulation in roots indicating that most S^0^ accumulates in the aerenchyma and most sulfate in the parenchyma. S^0^ seems unlikely to be present in the parenchyma. Roots and rhizomes exposed to high levels of sulfide can accumulate considerable amounts of S^0^, whereas S^0^ is not detectable in the leaves [6,11], suggesting that, even under high pore water sulfide concentrations coupled with hypoxic conditions and darkness, toxic gaseous sulfide does not reach the leaves. This also suggests that the sulfide-vulnerable leaf meristem, located at the basal part of the leaf, remains unexposed to sulfide. Similarly, Pedersen et al. [13] did not detect sulfide in the meristematic region of *Z*. *marina* at water column oxygen saturation levels of above 35%. The detoxification of sulfide to S^0^ in *Z*. *marina* supports previous studies proposing this mechanism [11,52,53] and we speculate that the precipitated S^0^ in the aerenchyma is permanently deposited and represents a sink of oxidized intruding sediment sulfide. Some terrestrial plants actively metabolize S^0^, which functions against fungal pathogens [25], but this is probably not the case for *Z*. *marina*, as the accumulation of S^0^ is due to passive chemical oxidation (by oxygen) rather than an active process. Further, the antifungal function seems unlikely because of the low impact of fungi in seagrass systems [54,55]. Thus, S^0^ presumably functions exclusively as a detoxification compound in underground tissues and can be used as an indicator of sulfide presence in the aerenchyma.

In contrast to S^0^, sulfate accumulated in both underground and above-ground tissues of *Z*. *marina* exposed to sulfide intrusion. Theoretically, three possible sulfate sources are present in seagrass beds: 1) sulfate derived from oxidation of sulfide in the leaves; 2) sulfate taken up from pore water and/or the water column; and 3) sulfate derived from oxidation of sulfide in underground tissue followed by translocation to the leaves. In this study, the first mechanism can be discounted due to the absence of sulfide in the leaves. Concentration- and demand-dependent uptake of sulfate from pore water and the water column can be discounted in this study due to the constant and high concentrations of the sulfur pools in all treatments; also, there is no expected increase in sulfur demand upon sulfide intrusion [56]. In addition, root sulfate uptake plays a minor role in plants exposed to gaseous sulfide [56] and we expect a similar lack of response for seagrasses. These findings favor the third hypothesis: that sulfate accumulation in *Z*. *marina* upon sulfide intrusion originates from direct oxidation of intruding sediment sulfide. This is further supported by the positive linear relation between pore water sulfide and tissue sulfate concentration, suggesting a positive coupling between sediment sulfide and plant sulfate accumulation. However, the excess sulfate derived from oxidized sulfide is probably surplus to demand and consequently transferred to vacuoles, where it can be stored [35]. Sulfate stored in the vacuoles might represent a possible end product of sulfide detoxification, similar to the function of S^0^.

### Incorporation of sediment sulfide

The increased thiol content in underground tissues of *Z*. *marina* indicates that intruding sulfides penetrating the tissue are incorporated into thiol compounds. Sulfide is probably enzymatically incorporated directly into cysteine and subsequently metabolized into organic sulfur compounds, as observed in terrestrial plants [32,57]. The thiols cysteine and glutathione represent important precursors of all organic sulfur compounds [58], feeding the synthesis of organic sulfur in *Z*. *marina* and pinpointing the relevance of the small but reactive thiol pool in controlling the sulfur metabolism in plants [59]. The thiol content in underground tissues increased linearly with sulfide concentration, suggesting no limitation of the sulfide incorporation mechanism under the experimental conditions. Similarly Westerman et al. [60] found linearity between atmospheric sulfide levels and the increase in thiol and cysteine concentration in the terrestrial plant *B*. *oleracea*. This linearity indicates a constant sulfide incorporation mechanism in *Z*. *marina* that cannot be controlled by the plant and that facilitates sulfide detoxification in seagrass.

The organic sulfur content increased in leaves, roots, and rhizomes upon sulfide intrusion and accounts for a substantial amount (29%) of the increase in total sulfur content. The accumulation of organic sulfur could be a result of thiol metabolism, thereby bypassing the “normal” assimilatory sulfur uptake pathway of sulfate via the roots or leaves. This suggests a possible role of sediment sulfide in the sulfur nutrition in Z. *marina*, as has been found for terrestrial plants exposed to sulfide fumigation [33,34].

### Stable isotope tracing

We traced the Δδ^34^S signal of sediment sulfide in the leaf, rhizome, and root tissues of *Z*. *marina* exposed to sediment sulfide, to assess the source of the accumulating sulfur compounds. Roots and rhizomes exposed to sulfide intrusion accumulated sulfur compounds characterized by a Δδ^34^S close to that of sediment sulfide, implying that sediment sulfide was the sole source of accumulated sulfur. Thus, a high share of the detoxification of sediment sulfide (86%) takes place in the underground tissues. In contrast, the leaves are characterized by a lower Δδ^34^S implying a lower share of sediment sulfide and thereby a lower contribution of the leaves to sulfide detoxification (14%).

The precipitation of sulfide as S^0^ is by far the dominant detoxification mechanism (67%), avoiding sulfide intruding from the aerenchyma into parenchymatous tissues. The remaining sulfide (33%) radially intruding from the aerenchyma into parenchymatous tissue is seized by a tolerance mechanism in which sulfides are incorporated into thiol compounds followed by metabolism to organic sulfur. Avoidance and tolerance mechanisms act in concert to avoid intruding sediment sulfide reaching the vulnerable leaf meristem.

Organic sulfur and S^0^ formed in the underground tissues upon sulfide intrusion are not translocated to the leaf, as indicated by the different Δδ^34^S of the tissues. In contrast, the similarity of roots, rhizome, and leaves in Δδ^34^S of plant sulfate accumulating upon sulfide intrusion implies that sulfate is the only compound that is translocated from underground to above ground tissues. This supports Rennenberg’s [35] suggestion that sulfate is the main sulfur transport form for plants in general. The mechanism lowers sulfur accumulation in underground tissues by translocating sulfate derived from toxic sulfide to the leaves, probably avoiding harmful hyperaccumulation of sulfate [35] in underground tissues.

**Fig 7:**
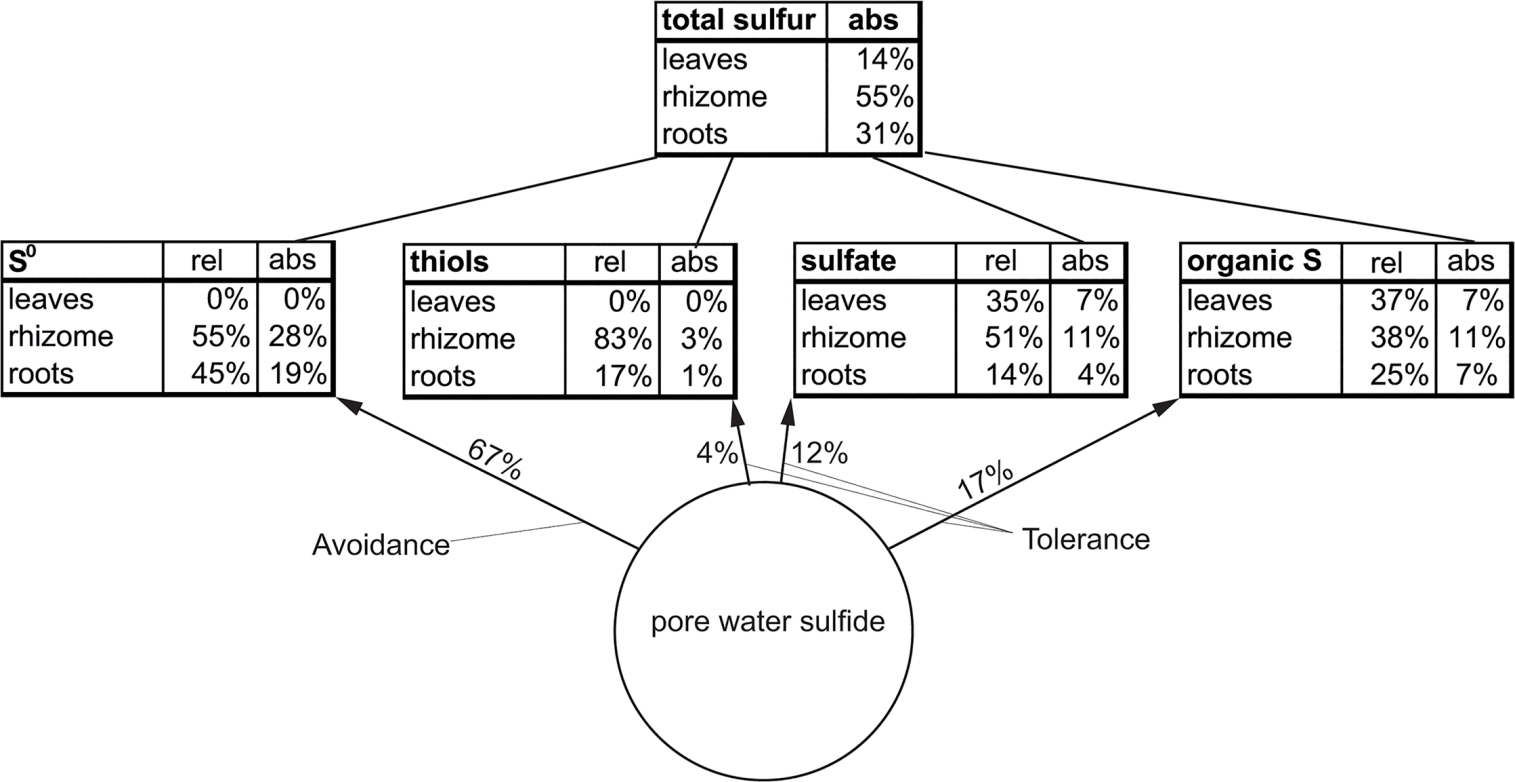
Conceptual model of sulfide detoxification capacities of *Zostera marina* exposed to sulfide intrusion based on stable isotope tracing and mass balance modeling. The numbers (percentage) next to the arrows indicate the contribution of a sulfur compound to the detoxification process. The numbers (percentage) under ‘rel’ indicate the relative distribution of a sulfur compound between the tissues; the numbers under ‘abs’ indicate the share of the net total sulfur accumulation upon sulfide intrusion of the compounds in a tissue. The total sulfur table indicates the share of the different tissues in detoxification of sulfide. Avoidance is indicated by preventing sulfide intrusion by precipitation of sulfide to S^0^ and tolerance by standing sulfide intrusion by incorporating sulfide into thiols, organic sulfur, and sulfate.

We propose a conceptual model of relative and absolute sulfide detoxification capacities, based on the contribution of sulfur sources to the different plant sulfur compounds after sulfide intrusion (Fig 7). It is noteworthy to mention that the mechanisms described above occur only upon high rates of sediment sulfide intrusion.

### Sulfur compounds and intrusion under natural conditions

Under natural conditions and moderate levels of pore water sulfide, roots accumulate the most sulfur, with the most negative δ^34^S, followed by rhizomes and leaves (Table 2) [24,52]. This indicates that sulfide intrusion is constant in the roots, occasional in the rhizomes, and non-existent in leaves, which is further supported by the presence of S^0^ in roots and rhizomes, albeit at low concentrations [24,52]. However, even at low sulfide levels, sulfide is incorporated and metabolized via thiols into organic sulfur. This is indicated by the δ^34^S signal of organic sulfur ranging in between those of sediment sulfide and pore water sulfate, suggesting mixed sulfur uptake from both sources. This suggests that under natural conditions—without hypoxia events—ROL is sufficient to protect the leaf meristem from sulfide intrusion. Moderate sulfide intrusion constantly happens during the night but the sulfide is completely detoxified in the roots and only marginal amounts reach the rhizome, without harming the plants. Although field studies are not directly comparable with mesocosm studies, the approximately five-times-lower sulfide concentrations in the field provide a lower sulfide pressure that most likely results in lower levels of sulfide intrusion. This may explain the lower amounts of sulfur compounds in the field plants, similar to previous findings in field plants [6,11,52].

### Detoxification pathway

**Fig 8:**
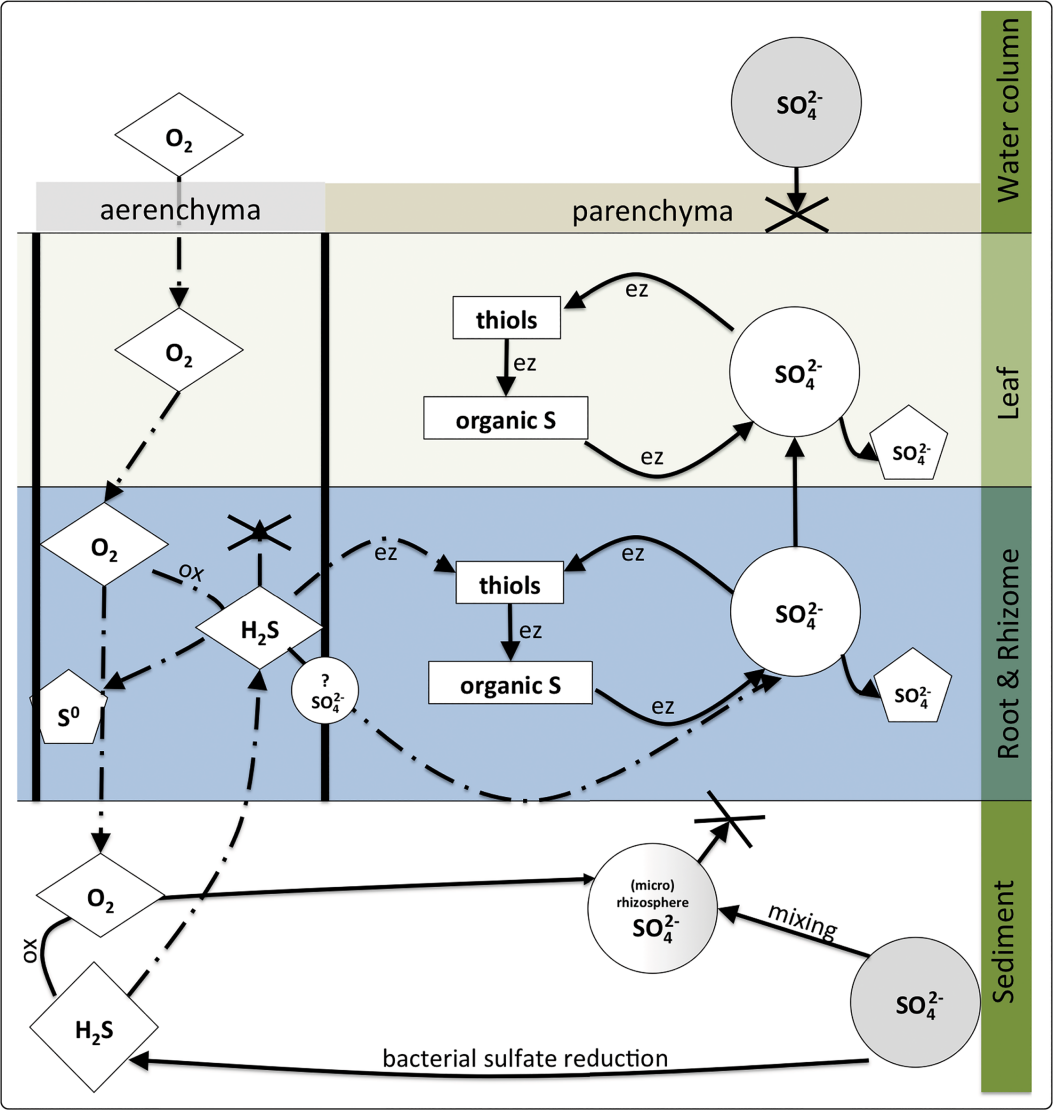
Conceptual model of sediment-sulfide intrusion, sulfate uptake, and possible sulfide accumulation/detoxification patterns in *Zostera marina*. Dash-dotted lines = gas diffusion; dashed lines = liquid diffusion; circles = inorganic sulfur; rectangles = organic sulfur; diamonds = gaseous compounds; pentagons = detoxified and permanently stored sulfur; ox = oxidation; ez = enzymatic pathway. Symbols marked with a question mark indicate an unknown reaction. Symbols shaded in grey indicate sulfur pools enriched in ^34^S. White symbols indicate depleted ^34^S sulfur pools and symbols with a grey/white gradient indicate mixed δ^34^S pools.

The results of our quantification of sulfur compounds and stable isotope tracing are summarized in a conceptual model in Fig 8. Sulfate in the pore water is reduced by sulfate-reducing bacteria to sulfide, which is depleted in ^34^S due to isotopic fractionation. The sulfide is subsequently oxidized to sulfate by ROL in the oxic halo around the roots; this process may be facilitated by sulfide oxidizing bacteria [20,61]. However the resulting sulfate has a δ^34^S similar to sediment sulfide, and is mixed with the bulk pore water pool of sulfate, resulting in a δ^34^S somewhere between those of sulfide and sulfate. During events of hypoxia and darkness ROL decreases and sediment-derived sulfide may intrude into the aerenchyma of roots and rhizomes. The intruding sulfide is chemically oxidized by oxygen to S^0^, precipitating on the inner wall of the aerenchyma, or oxidized to sulfate. The remaining sulfide radially penetrates the parenchymatous tissue and undergoes enzyme-facilitated incorporation into thiols. Thiols are further metabolized into organic sulfur or oxidized to sulfate. Thus, the oxidation of sulfide to sulfate occurs either in the aerenchyma or inside the tissue. Sulfate formed in the underground tissues is stored or translocated to the leaves, where it is stored and/or fuels sulfur metabolism. Under oxic water column conditions, the detoxification mechanisms prevent the intruding sulfide from penetrating the meristematic region in the leaf, whereas under anoxic or hypoxic conditions the sulfide may enter and lead to toxicity.

## Conclusions

Seagrasses can thrive in sulfidic sediments due to two major detoxification strategies: avoidance, by oxidation of sulfide; and tolerance, by incorporation of sulfide into plant tissues. Sulfide is oxidized and precipitated as S^0^ in the aerenchyma or is oxidized to sulfate inside or outside the plant. Further sulfide is incorporated as thiols and sulfate. S^0^ and thiols are stored in rhizomes and roots, whereas sulfate is transported from under- to above-ground tissues. Underground tissues possess the highest detoxification capacity (86%), and the rhizome (61%) acts as the primary buffer in detoxifying toxic sediment sulfide and protecting the vulnerable meristematic tissue in the leaves. Future climate scenarios predicting higher surface water temperatures [62], higher frequencies of hypoxic events [63] and increasing sediment sulfide levels [64] will lead to higher sulfide pressure on seagrass ecosystems. Such conditions might exceed the sulfide tolerance and detoxification capacities. Our findings open a discussion of how seagrasses cope with sulfidic sediments in future climate scenarios and how detoxification mechanisms vary between seagrass species and growth stages.

## Supporting Information

S1 Fig Linear regression of Δδ^34^S of sediment sulfides and Δδ^34^S of different fractions of *Zostera marina* tissues exposed to high levels of sulfide intrusion; note different scales on the y-axes. R^2^, p, and the linear regression equation are presented next to the plot. asnf = assumptions for linear regression not fulfilled. n = 6.
(DOCX)

S1 Table Pore water nutrients and sulfide levels [mean ± SEM] in sediments enriched with glucose matter (HS) and control, sampled weekly for 4 weeks. n = 12.
(DOCX)
