# Supplementary material for: Sulfide Intrusion and Detoxification in the Seagrass Zostera marina
Source: PLoS One. 2015 Jun 1;10(6):e0129136. doi: 10.1371/journal.pone.0129136 (PMC4452231; doi:10.1371/journal.pone.0129136)
Supplement: S1 Fig — R2, p, and the linear regression equation are presented next to the plot. asnf = assumptions for linear regression not fulfilled. n = 6. (DOCX) [file pone.0129136.s001.docx]

|  | **bulk** | **S^0^** | **organic S** | **sulfate** |
| --- | --- | --- | --- | --- |
| **R^2^** | asnf | asnf | 0.9126 | 0.7836 |
| **p** |  |  | 0.003 | 0.019 |
| **equation** |  |  | 1.1*x - 17.9 | 2.4*x - 44.2 |

|  | **bulk** | **S^0^** | **organic S** | **sulfate** |
| --- | --- | --- | --- | --- |
| **R^2^** | 0.8994 | 0.8854 | 0.8498 | 0.9266 |
| **p** | 0.0039 | 0.0051 | 0.0089 | 0.0021 |
| **equation** | 1.8x-22.4 | 1.1x-9.8 | 1.7x-22.2 | 2.1x-34.5 |

|  | **bulk** | **S^0^** | **organic S** | **sulfate** |
| --- | --- | --- | --- | --- |
| **R^2^** | 0.6976 | 0.8654 | 0.7458 | 0.6617 |
| **p** | 0.0385 | 0.0071 | 0.0266 | 0.049 |
| **equation** | 1.6x-22 | 1.5x-21.5 | 1.5x-21 | 1.9x-24 |

**S1 Figure: Linear regression of Δδ^34^S of sediment sulfides and Δδ^34^S** of different fractions of *Zostera marina* tissues exposed to high levels of sulfide intrusion; note different scales on the y-axes. R^2^, p, and the linear regression equation are presented next to the plot. asnf = assumptions for linear regression not fulfilled. n=6.
