# Supplementary material for: Sulfide Intrusion and Detoxification in the Seagrass Zostera marina
Source: PLoS One. 2015 Jun 1;10(6):e0129136. doi: 10.1371/journal.pone.0129136 (PMC4452231; doi:10.1371/journal.pone.0129136)
Supplement: S1 Table — n = 12. (DOCX) [file pone.0129136.s002.docx]

**S1 Table: Pore water nutrients and sulfide** [mean ± SEM] in sediments enriched with glucose matter (HS) and control, sampled weekly for 4 weeks. n = 12.

|  | **Sulfate [mM]** | **Sulfide [µM]** | **Ammonium [µM]** | **Phosphate [µM]** |
| --- | --- | --- | --- | --- |
| HS | 14.40 ± 1.63^a,A^ | 2845 ± 378 ^a,A^ | 214 ± 34.85 ^a,A^ | 24.6 ± 1.9 ^a,A^ |
| Control | 14.05 ± 1.32 ^a,A^ | 864 ± 303 ^b,A^ | 241 ± 44.86 ^a,A^ | 25.3 ± 2.1 ^a,A^ |
|  |  |  |  |  |

Different lower case letters indicate significant differences between the treatments and upper case letters between time (ANOVA p < 0.05, Tukey test).
